# Forest work and its implications for malaria elimination: a qualitative study

**DOI:** 10.1186/s12936-019-3008-3

**Published:** 2019-11-27

**Authors:** Nou Sanann, Thomas J. Peto, Rupam Tripura, James J. Callery, Chea Nguon, Thanh Mai Bui, Stephanie D. Nofal, Lorenz von Seidlein, Dysoley Lek, Arjen M. Dondorp, Phaik Yeong Cheah, Christopher Pell

**Affiliations:** 1 Mahidol Oxford Tropical Medicine Research Unit, Faculty of Tropical Medicine, Mahidol University, Bangkok, Thailand; 2 University Research Company, Phnom Penh, Cambodia; 3 Centre for Tropical Medicine and Global Health, Nuffield Department of Clinical Medicine, University of Oxford, Oxford, UK; 4 National Center for Parasitology, Entomology, and Malaria Control, Phnom Penh, Cambodia; 5 Faculty of Health, Medicine and Life Sciences, Maastricht University, Maastricht, The Netherlands; 6 Faculty of Infectious and Tropical Diseases, London School of Hygiene and Tropical Medicine, London, UK; 7 Amsterdam Institute for Global Health and Development, Amsterdam, The Netherlands; 8 Centre for Social Sciences and Global Health, University of Amsterdam, Amsterdam, The Netherlands

**Keywords:** Malaria, Elimination, Forest, Greater Mekong Sub-region, Qualitative

## Abstract

**Background:**

Over the last 20 years, malaria incidence has decreased across the Greater Mekong Sub-region (GMS) and the emergence of artemisinin resistance has stimulated efforts to accelerate regional elimination. In the GMS, the malaria transmission is focused increasingly in forested zones. This article describes forest-going activities and examines forest workers’ attitudes to and experiences of malaria prevention and control in north-eastern Cambodia.

**Methods:**

In Stung Treng Province, Cambodia, 19 in-depth interviews were conducted in villages with participants recently diagnosed with uncomplicated falciparum malaria who reported working in forests. Two focus group discussions with respondents’ forest-working peers were held. Interviews and focus groups were audio-recorded transcribed, and translated for thematic analysis.

**Results:**

Forest work is an essential source of income for respondents. Many combine it with farming, which influences the timing and duration of forest visits. Forest activities include logging and collecting other forest products, particularly malva nuts. Men log year-round, whereas gathering forest products is seasonal and can involve entire families. Forest workers sleep chiefly in unimpregnated hammock nets in make-shift encampments. Respondents are concerned about symptomatic malaria, but unfamiliar with the concept of asymptomatic infection. They view the forest as an area of potential malaria infection and seek to protect themselves from mosquito bites through wearing long-sleeved clothes, using repellents, and lighting fires. Forest workers express a willingness to self-test and self-administer anti-malarials.

**Conclusions:**

Forest workers’ behaviour and perceptions of risk indicate that improvements are needed to current control measures. There is potential to: better target distribution of impregnated hammock nets; offer curative or presumptive treatment while in forests; and expand access to screening. Establishing the efficacy and feasibility of prophylaxis for forest workers in the GMS is a priority.

## Background

Since the turn of the century, the Greater Mekong Sub-region (GMS) has reported a large decrease in malaria incidence and related mortality [1]. Drawing on increased funding, the region has seen notable achievements in malaria prevention and control: improved vector control, enhanced case detection and greater availability of effective anti-malarial treatments [2]. Regional and national economic processes, including changes in land-use, have also played a role [3].

In recent years, progress towards elimination has not been smooth and was even temporarily reversed in some locations, such as Cambodia, where incidence rose from 2016 to 2017 [4]. The public health importance of elimination is amplified by the emergence and spread of multidrug resistant *Plasmodium falciparum* strains in the region [5–7]. With no alternative drugs to replace artemisinin-based combination therapy (ACT) as first-line treatment, this could have severe consequences beyond the region, particularly were resistant parasites to spread to sub-Saharan Africa [8, 9]. In the context of these developments, governments of the GMS have set themselves the goal of eliminating malaria from the region by 2030 [1, 4].

In the GMS, malaria parasite reservoirs cluster along international borders and around forests [10–12]. In these areas, malaria remains endemic in high-risk populations, including mobile migrant workers and forest workers [13–15]. With infections often acquired outside villages, forest workers are at high risk of *P. falciparum* and *Plasmodium vivax.* Forest workers are also at risk of zoonotic malaria because they come into contact with macaques and other monkey species, which carry simian *Plasmodium* species, such as *Plasmodium cynomolgi* and *Plasmodium knowlesi* [16].

A recent systematic review of qualitative research on malaria-related practices and attitudes of forest-goers in the region highlighted a range of factors that put them at increased risk of malaria: the limited protection offered by current vector control interventions, such as insecticide-treated bed nets and indoor residual spraying (IRS), because the major malaria vectors bite outdoors and during daytime; and the limitations of village-based approaches that do not specifically include people who are working in forests [17]. However, there is a lack of research on the nature of forest activities, and the need to understand forest work to better tailor intervention packages [17].

Drawing on in-depth interviews (IDIs) and focus group discussions (FGDs) with forest workers in Siem Pang District, Stung Treng Province, north-eastern Cambodia, this article aims to identify how malaria elimination programmes can be tailored to forest workers’ activities, needs and preferences. The article describes forest work and forest workers’ attitudes to and experiences of malaria prevention and control tools, and examines the practical challenges of targeting forest workers within malaria elimination efforts.

## Methods

### Setting

**Fig. 1:**
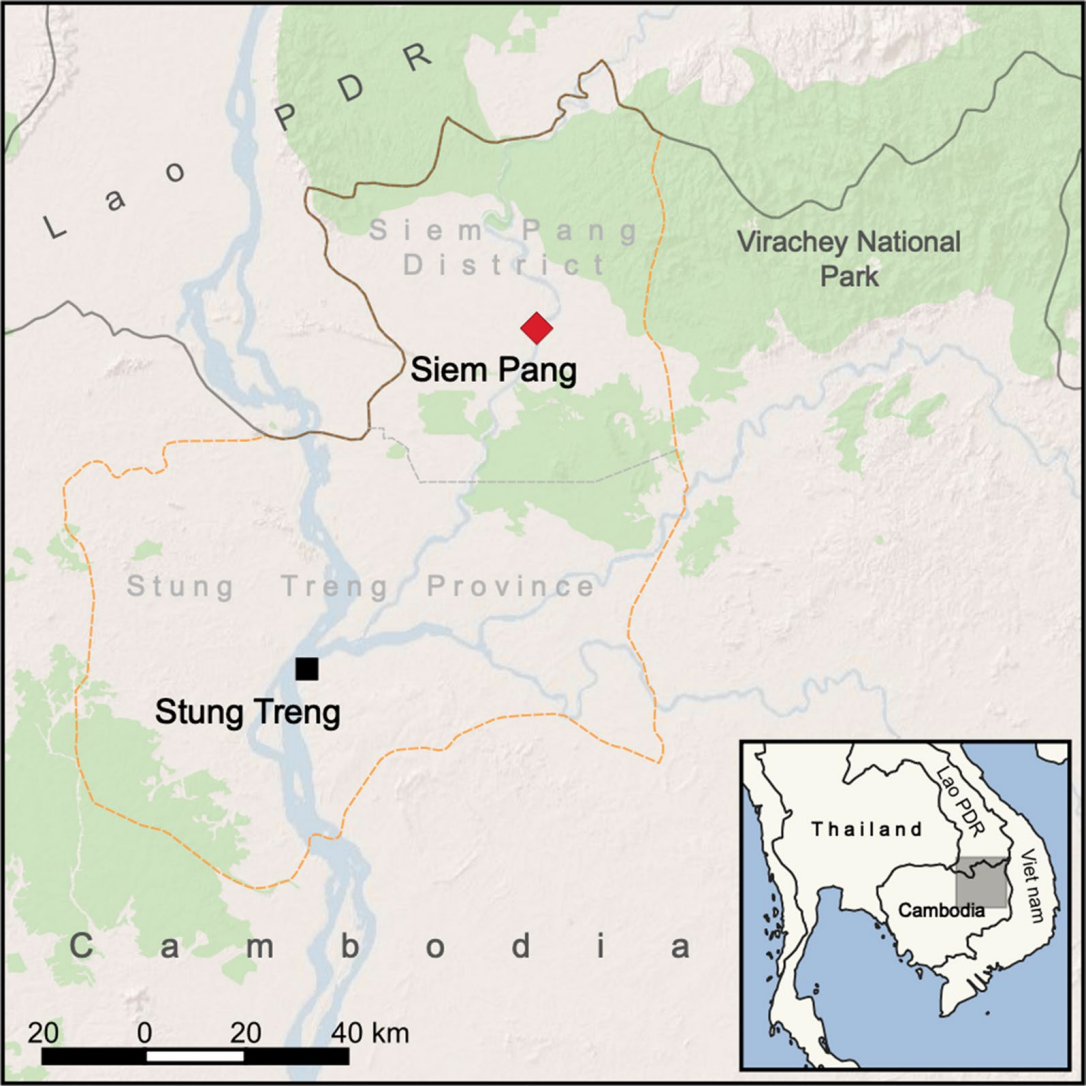
Map of Stung Treng Province

**Fig. 2:**
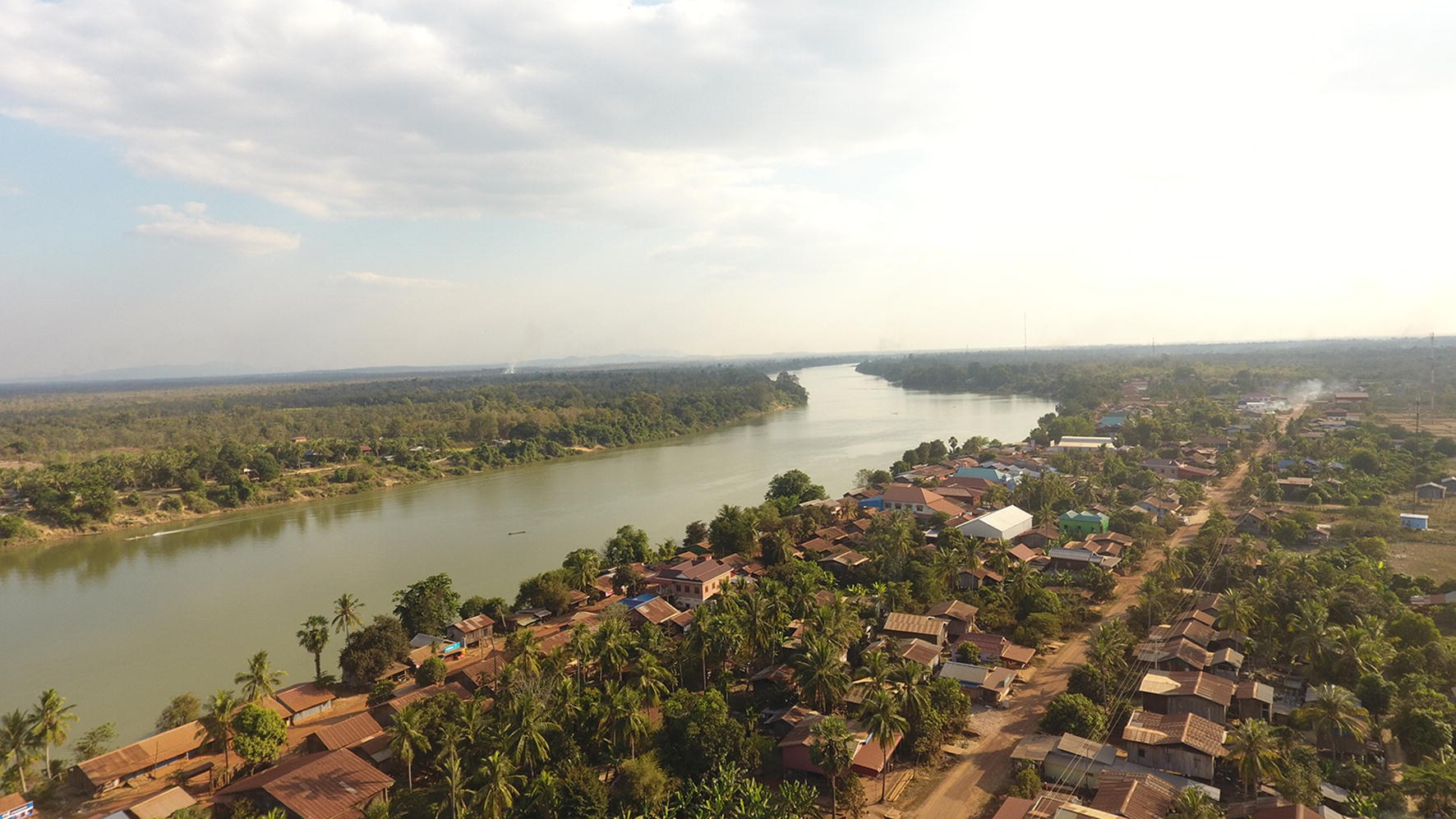
Aerial image of Siem Pang town and surroundings

Stung Treng Province, located in north-eastern Cambodia, borders Lao PDR to its north and west (Fig. 1). The province is bisected by the Mekong River and is predominantly rural. With over 14,000 cases reported in 2014 [18], Stung Treng has among the highest incidence of malaria in the country, with Siem Pang District recognized as an area of intense transmission. The Sekong River (also known as the Tonle Kong) flows through Siem Pang and seasonal heavy rains can severely affect road access to villages in the surroundings. Siem Pang has a diverse population that includes ethnic minority groups, including Laotian and Kaviet groups. Farming is the main livelihood activity. Forest work is also evident in the area, with logging particularly noticeable. There are densely forested areas in the western part of Virachey National Park and surrounds, which lie to the north of the district close to the border with Laos (Fig. 2). Cross border population movement can present challenges to malaria control and there is little systematic description of cross-border movements in this area.

### Respondents

As part of a clinical trial evaluating the efficacy of new anti-malarials for *P. falciparum* (ClinicalTrials.gov Identifier: NCT03355664 [19]), based at the Siem Pang Health Centre, data on risk factors for malaria infection were collected routinely by questionnaire from participants. In-depth interviews were conducted with clinical trial participants who reported visiting forested areas in the six months prior to their most recent malaria infection. Following a participant’s recovery and completion of the 42-day follow-up period in the clinical trial, potential respondents were approached by members of a community engagement team attached to the clinical trial to ask about forest-related activities. Focus group discussions were conducted with respondents recruited through snowball sampling to identify other community members who visit forests. Recruitment took place in villages surrounding Siem Pang, where previous activities had taken place to inform local communities about the study underway in the health centre.

### Data collection

Data collection tools—an IDI and FGD guide—were developed based on the initial topics of interest drawn from a recent review of research on the forest going and malaria related risk in the GMS [17] and issues identified by field staff at Siem Pang Health Centre: the nature of forest work (activities, timing, itinerary, group composition), familiarity with malaria (including the possibility of asymptomatic malaria), malaria diagnosis and treatment, and attitudes to malaria prevention and control measures (prophylaxis, vaccination and self-treatment). Initial IDI and FGD guides (see Additional file 1: Annex S1) were designed in English and translated by a native Khmer speaker and a social scientist/field researcher with 20 years of experience. The guides, which included key topic areas and lists of suggested questions, were designed to be used in a flexible and iterative manner: interviewers would use the appropriate questionnaires to elicit information on the specific topic of interest. The translation of the topic areas and suggested questions were discussed with the team who were also trained on how to use the guide. The guide was then piloted with the first recruited study participants to check for miscommunications and revised as necessary.

Interviews took place in villages, typically near to participants’ homes. All of the villages lie within Siem Pang District and within the catchment area of Siem Pang Health Centre and typically close to roads and the river. Respondents were interviewed by one of three trained field researchers fluent in Khmer. Interviews took place in Khmer. In the case of respondents experiencing difficulties in expressing themselves in Khmer, translation (usually only of specific terms) was sought from other study staff members who were fluent in Laotian or one of the local languages.

### Data processing and analysis

With the consent of respondents, interviews were audio-recorded and subsequently transcribed verbatim and translated to English by professional transcribers/translators. The translations were checked by one of the interviewers (a bilingual native Khmer speaker and experienced field researcher), who listened to the audio-recordings alongside the translated transcripts to identify inconsistencies. The checked translations were imported into NVivo version 11 (QRS International, Australia) for qualitative content (thematic) analysis. All transcripts were read several times and coded line-by-line using an inductive and deductive approach: the codebook used was initially based on the main research topics. For example, codes were included to capture the different dimensions of forest activities (including location, timing, nature). Subsequently, during the process of coding, themes that emerged from the data were incorporated into the codebook. Patterns across responses were identified for example, regarding the impact of village location or ethnicity on the nature of forest work or experiences with malaria treatment.

### Ethics approval

In Cambodia, approval was obtained from the National Ethics Committee for Health Research (NECHR, reference 210, renewed 23rd February 2018) and from the Oxford Tropical Medicine Research Ethics Committee (OXTREC, approval reference: 32-17, 30th November 2017). All respondents provided informed consent to participate in the study and for the interviews to be audio-recorded. Written informed consent was obtained from all study participants when they were recruited to participate at the Siem Pang Health Centre and subsequently consent was re-obtained verbally (and audio-recorded) immediately before the interviews were conducted in their villages several weeks later.

Local authorities and other communities were engaged from an early stage of the clinical trial and the ancillary qualitative research. Before beginning data collection, meetings with local authorities were held to explain the activities and their purpose. Subsequently, major engagement activities were held in study villages to familiarize communities with the research group. There was regular contact between the study team, local health workers and village, commune and district-level leaders.

## Results

In-depth interviews were conducted with 19 male respondents, drawn from villages in Siem Pang District and with one from Siem Pang town. The respondents were between 16 and 51 years and identified as Khmer, Kaviet, Khe, and Laotian (Table 1). Two focus groups were conducted with forest-goers: one in Lakay village with six male respondents aged 17 to 51 (who identified as Khmer); and one with eight male respondents aged 21 to 44 and drawn from villages around Teak Team (who identified as Kaviet). Men were not recruited specifically but all respondents were male because of the nature of forest work.

**Table 1:** Respondent characteristics

Village	Age group	Mother tongue(s)
Ban Muong	51–55	N/A
Lakay	16–20	Khmer
Khan Doeur	N/A	Khmer
Kiribas Leu	16–20	Kaveit
Kiribas Leu	16–20	Kaveit
Kiribas Leu	16–20	Kaveit
Kiribas Leu	16–20	Kaveit
Kiribas Leu	26–30	Kaveit
Lakay	16–20	Kaveit
Lakay	16–20	Khmer
Lakay	16–20	Khmer
O Chay	N/A	Kaveit
Samor	16–20	Lao/Khmer
Samor	16–20	N/A
Samor	31–35	Khmer
Samor	36–40	Lao
Siem Pang	26–30	Khmer
Thmor Keo	21–25	Khmer
Toul Veng	21–25	Khe/Lao/Khmer

The findings describe the nature of the reported forest activities, and malaria prevention and control-related practices. Attitudes to malaria prevention and control interventions are described. The findings are based on the IDIs and FGDs, with quotations used to illustrate the issues raised in the (translated) terms of the respondents.

### Forest visits

#### Reasons for visiting forests

The respondents visited forested areas to supplement their income from farming or as their main livelihood activity. Focus group respondents cited poverty and a lack of economic alternatives as their reason for working in the forests. The main income-generating activities were collecting wood/logging (mentioned by 13 interview respondents) and collecting forest products, particularly malva nuts. The latter was described as a seasonal activity, whereas logging was undertaken year-round, with some variation depending on access issues (further described below). Six respondents mentioned hunting and three referred to fishing as forest-based activities. For a couple of respondents, hunting and fishing were used to generate income but for most they were a way to provide sustenance for their time away from the village.

- *Interviewer [I]: Why do you have to go to the forest?*
- *Respondent [R1]: For our livelihood. [We have] no other source of income besides going to the forest.*
- *I: [R2], may I know why you go to the forest?*
- *R2: Because I am poor.*
- *I: How about you, [R3]?*
- *R3: I am, too.*
- FGD in Lakay Village

- *I: What do you do at the forest?*
- *R: I collect woods or hunt animals like monitor lizards*
- IDI in Toul Veng village

#### Forest destinations

Respondents’ forest destinations and itineraries depended on their livelihood, particularly whether they combined forest-based activities with farming. In general, respondents described journeys of around a day or more to reach the locations where they log based on the availability of the product of interest. Depending on the distance between locations in the forest, respondents described maintaining a single camp or moving camp.

*Unlike farming which allows us to cultivate at the same place, we have to go to a new area when no more products are available in the same area of the forest.*

IDI in Lakay Village

#### Frequency and length of forest stays

The total time spent in forested areas varied depending on the nature of activities, from several days to 6 months, with a frequency of 2 to 20 times per year. The dry season was considered by some as the more popular time to visit the forest because roads were less muddy and more easily passable than during the rainy season.

- *I: How many days do you usually spend at the forest?*
- *R1: Yes. I usually stay in the forest, mostly, for a week. And one month if I have to stay longer. And I go once … well, I do sleep under a bed net or in a hammock net, but I still get malaria.*
- *R2: It depends. Sometimes, I stay there for one or two months.*
- *I: …Why is it so long?*
- *R2: Because the tractor may be broken, or we to come back and forth to the village when we run out of food.*
- *R3: Yes. I go to the forest … Like him, sometimes it also takes one month. If it doesn’t take long, it will be half a month only.* 
- *R4: I rarely go to the forest. I stay there for only four or five days.*
- FGD in Teak Team village

#### Who are the forest workers?

Respondents described forest work as always communal. The composition of the groups who visited the forest together varied with the activity, distance and means of transport. Logging was an activity undertaken by groups of men, usually friends or relatives from the same village. Collecting malva nuts often drew mixed-gender groups, with husbands and wives sometimes working together. Groups were important when traveling by hand-tractor, to push the vehicle across the steep or muddy terrain. When in the forests, respondents often came across other groups engaged in similar activities.

- *I: Do you see other groups of forest goers?*
- *R1: Yes.*
- *I: How many people are there in each group?*
- *R1: three or five people. Or sometimes six or seven.*
- *I: Mostly, are they women or men?*
- *R1: Mostly, men.*
- *I: Have you seen women going to the forest?*
- *R1: No.*
- *R2: They go to the forest during malva seasons.*
- *I: From what age are those women?*
- *R3: Even a baby…haha… [all laugh]*
- *R4: The indigenous people even take their toddlers with them.*
- *I: Everyone including the little children?*
- *R4: All family members will be there. Unlike us, we don’t do the same …*
- FGD in Lakay village

#### Daily routine

Forest work was influenced by the seasons, specific activities and personal preference. Work began between 4 a.m. and 8 a.m.; and ended between 7 p.m. and 11 p.m. In contrast, one respondent explained how he went hunting at night time and slept during the day. Evening times were used for socializing, with only one respondent reporting regular alcohol consumption in the evening.

- *I: What time do you sleep in the forest? From what time to what time?*
- *R1: It depends on the situation. At 7 or 8* *p.m. in the dry season, whereas, in the rainy season, we may finish work late at 8 or 9* *p.m., when we have to cook as well.*
- *I: When do you get up?*
- *R1: Some may get up at around 4* *a.m. while others get up at 7 or 8* *a.m.*
- FGD in Teak Team village

- *I: What time do you usually go to sleep when you are in the forest?*
- *R: At 6 p.m.*
- *I: What time do you wake up?*
- *R: At 5 a.m.*
- *I: Have you ever stayed late to eat and drink with others until 10 p.m?*
- *R: Yes.*
- IDI in Kiribas Leu

#### Sleeping arrangements

Sleeping arrangements in the forests typically involved temporary camps, using individual hammocks and plastic tents or sheets to provide cover. Typically, groups would arrange their hammocks close to one another. The presence of water nearby influenced the choice of campsite location. Hammocks used by respondents usually incorporated a mosquito net. These were not necessarily insecticide-impregnated, referred to as “American” nets and bought from local markets. In contrast, respondents described that when in the village they used regularly the insecticide-treated bed nets that had been distributed by health centres and village malaria workers (VMWs).

- *I: Could you describe your sleeping area? How do you usually arrange it? Are there any cover or walls?*
- *R: There are no walls but a cover which I can feel the cool breeze.*
- *I: How many people are there in your group?*
- *R: There were six people when I went to O’ Ta Ngor while fewer men went to Samrong. Sometimes, there are changes among the group members at different times.*
- *I: Do you sleep near other members of your group in the forest?*
- *R: Yes, we do. We sleep in separate hammock nets and huts.*
- IDI in Lakay village

### Malaria prevention and treatment

#### Recognizing malaria infection

Prior to the bout of malaria for which they were enrolled into the clinical trial, all respondents described previous experiences with malaria. For some, it was a regular occurrence:

- *I: Have you got malaria before?*
- *R: Yes.*
- *I: How many times until now?*
- *R: Too many times that I can’t remember…About 100 times. Six times a year or even twice a month.*
- IDI in Lakay Village

Although respondents explained that they were generally able to distinguish malaria from other febrile illnesses, there was some variability and vagueness in the symptoms that they associated with the disease: fever, chills, vomiting, headache, eye pain/dryness, bitter taste, exhaustion, lack of appetite or thirst, dizziness, hot flushes, generalized/muscle pain, paleness, malaise, fatigue and cold feet. Respondents cited different species of malaria—with vivax and falciparum mentioned—but made no distinction in terms of symptoms (or other implications).

- *I: What are the symptoms [of malaria]?…*
- *I: I would have a headache. I would feel pain in my bones, my arms and my feet.*
- IDI in Kiribas Leu village
- *I: Because each of you here has experienced malaria, I’d like to ask you about its symptoms.*
- *R: Sometimes, we might have dry eyes, a headache and shivering. Also, I get an unpleasant, bitter taste in my mouth.*
- FGD in Lakay village

These symptoms prompted respondents to seek diagnosis from the VMW or at a (public) health centre or private clinic. At any of these locations, a rapid diagnostic test (RDT) for malaria was viewed as necessary to make a formal diagnosis and for drugs to be prescribed. Respondents were generally familiar with RDTs and, when asked, most reported that they trusted the test result. When asked about the possibility of having malaria without symptoms, most were unconvinced and some confused by this idea, particularly when they had been asked about the symptoms associated with malaria infection. Three were open to the idea, though little explanation was offered.

- *I: Earlier we discussed the symptoms of malaria, but do you think it’s possible to be infected with malaria but not have any symptoms? There is no headache nor cold, yet could you have malaria?*
- *R1: I’m not sure about this.*
- *I: Do you understand the question? You know that you have the disease when you have headache and get a cold. What if you don’t have these symptoms? Could you have malaria?*
- *R1: If there are no symptoms, I think it’s not malaria.*
- IDI in Lakay Village

- *I: Do you think it’s possible to get malaria but not have any symptoms?*
- *R: I usually have some symptoms.*
- *I: What I mean is you usually have high temperature or headache, which makes you do the blood test for malaria. But, now I don’t have any symptoms and look healthy, do you think it’s possible I am having malaria?*
- *R: I don’t know. I think I know it when I’m not well.*
- *I: How? Do you mean when you have symptoms?*
- *R: Yes.*
- *I: Symptoms refer to when you have headache or dizziness. However, you don’t have these symptoms. Could you be infected with malaria?*
- *R: No.*
- IDI in Kiribas Leu village

The respondents associated malaria with forest visits because of the presence of mosquitoes. They viewed malaria infection in their villages as possible, but less likely because there are fewer mosquitoes. Some were able to name specific places where malaria was an issue, generally associated with the environmental conditions. Malaria was described as one of the hazards of forest work that could not be avoided because of the economic imperative of this work. Respondents were aware of the limitations of their protective measures because they were bitten in the daytime, despite wearing long clothing.

- *I: Can you think of the areas that is the most affected [by malaria]?*
- *R1: Nowadays, most people who get malaria are those who go to O Thmor Rolouy or O Dok Puet areas. Because a lot of them migrate there, so does malaria.*
- *I: Are they the areas of thick forests or mountains?*
- *R1: Both thick forests and mountains.*
- FGD in Lakay Village

#### Protecting oneself from malaria in the forest

Respondents described using hammocks with integrated mosquito nets as a way of protecting themselves from malaria whilst in the forest. They acknowledged that sometimes—after an exhausting day of work—they fall asleep and forget to hang the net. Some of the hammock nets that were described as non-impregnated were bought from the local markets. They mentioned wearing long-sleeved clothes to prevent bites but complained about the heat and being bitten by mosquitoes even when wearing such garments. Some respondents described using repellents, mainly mosquito coils at night. Lighting a fire was occasionally seen as a way of preventing mosquito-bites.

- *I: Do you know how to prevent mosquito bites?*
- *R: We should wear long sleeves and use bednets.*
- *I: Can you think of anything you didn’t do that put you at risk of getting malaria?*
- *R: I got mosquito bites while I was working wearing a short sleeve shirt.*
- IDI in Samor village

- *It’s very hot in the dry season, but we mostly use [the hammock] in the rainy season. [But] mosquitoes don’t bite us during that time: usually, we get bitten whilst working*
- FGD in Teak Team village

Respondents did not mention taking drugs prophylactically (whether for malaria or other infections). Moreover, their understanding of vaccination as a means to prevent disease in general was very poor: some mentioned a couple of diseases that are prevented by vaccinations, others were completely unaware of vaccination and many described not having ever been vaccinated. When asked about the possibility of taking medicines to prevent malaria, there were mixed responses: for some, they did not see them as necessary or did not like taking medication in general, whereas for others, the risk of malaria was such that taking medicines could be justified. For one respondent, this was on the assumption that the preventive drugs did not have any side effects.

*If the medicines could surely prevent malaria, I’ll take them. On the contrary, I won’t take the medicines if they are the same as the ones for treating malaria. I’m concerned they will affect my health.*

FGD in Teak Team village

#### Treating malaria

Respondents were familiar with the VMWs and described them as the first point-of-call if they suspected malaria infection. Two respondents however mentioned having encountered shortages of drugs that made them seek care at a (government-run) health centre or private clinic. Most mentioned blood tests—from the VMW or the health centre—as the means to confirm an infection. Most had received a positive RDT diagnosis in the past and had taken anti-malarials. They viewed the RDT result as accurate, although one respondent had doubts after one instance of a negative RDT yet positive microscopy result (at a private clinic). Respondents linked some complaints to the anti-malarials but reported that unwanted effects would not influence whether they took anti-malarials in the future to treat a bout of malaria. Two respondents described carrying RDTs, bought from the market or acquired from a VMW, into the forest in case any member of the group fell ill, as a means of determining whether they had to seek assistance. Four others described taking paracetamol on forest visits to treat the symptoms of minor febrile illnesses. During the focus group held in Lakay village, the possibility of taking anti-malarials for treatment purposes was raised and respondents were keen to “be prepared in advance”.

- *I: Have you ever been tested and get a negative result when you were sick?*
- *R1: Yes.*
- *I: Yes?*
- *R1: Yes, one time I felt a bit dizzy and a bit cold, so I went to see the VMW. She told me that I didn’t have malaria, but flu. She gave me medicines and I felt better. Sometimes I had gastroenteritis. I was given medicines and I recovered.*
- *I: So do you trust the RDT results to be accurate?*
- *R1: Yes.*
- *I: Did you bring it with you to the forest?*
- *R1: Yes.*
- *I: Who gave it to you?*
- *R1: I bought it at a market.*
- *I: How many did you bring with you each time you went to the forest?*
- *R1: two or three RDTs.*
- IDI in Sean Moeur village

## Discussion

The findings offer insight into practices and attitudes that put forest workers at risk of malaria infection. These are essential considerations for the design of prevention and control strategies for forest workers from culturally diverse villages in north-eastern Cambodia. Research on this topic—and the tailoring of interventions—is crucial because forest workers form a priority population for the elimination of malaria in the GMS [13]: they are at greater risk of sub-clinical infections [20] and their use of preventative interventions is often sub-optimal [17]. Infection outside villages brings a greater risk of *P. vivax*. Forest workers are also at risk of zoonotic malaria because they are more likely to come into contact with macaques and other monkey species, which carry predominantly simian *Plasmodium* species, such as *P. cynomolgi* and *P. knowlesi*, known to be capable of infecting humans [16].

The findings underline how—in the villages of Siem Pang, as in other rural settlements close to forests in the GMS [21]—forest work is part of everyday lives and remains an essential source of income. Respondents spent time in the forests undertaking a complex mix of activities, including logging and collecting other forest products, particularly malva nuts: logging was undertaken year-round by groups of men; whereas gathering forest products was more of a seasonal activity in which whole families could participate. These activities were not mutually exclusive and were sometimes combined with hunting and fishing. For some, forest work was their only source of income, whereas others combined it with farming, which impacted the timing and duration of forest visits. Daily routines in the forest varied, depending on the nature of activities, seasons and personal preference. Such heterogeneity complicates the tailoring of a malaria intervention package aimed at forest workers.

There were notable similarities across the respondents, for example, sleeping in hammock-nets often not impregnated with insecticides. Most undertook their forest visits in groups and stayed in make-shift encampments with hammocks close to one another. They often encountered other groups in the forest, increasing the risk of malaria transmission. In general, respondents were concerned about malaria, and—as elsewhere in Cambodia and the wider GMS [22–27]—viewed the forest as an area of potential infection and sought to protect themselves—often sub-optimally—from mosquito-bites and malaria through wearing long-sleeved clothes, using mosquito repellents and/or lighting a fire.

In terms of vector control measures, the tendency to use non-insecticide-impregnated hammock nets indicates at least one opportunity for improved prevention and control (here and elsewhere in Cambodia [28, 29]). Because forest workers can easily be neglected in village-based approaches [22, 23, 30], the distribution of impregnated hammock nets would ideally be targeted toward members of forest-going groups or family members who could—if necessary—distribute nets to others who visit forests. In this area, distribution should not take place during the malva nut season when many community members are likely to be absent from villages.

The willingness of forest workers to carry medicines with them to the forest to treat febrile illness [27, 31] suggests some form of self-administration of an anti-malarial might be acceptable. Many respondents considered a set of symptoms as “malaria”, with any underlying infection confirmed by RDT. They were unsure about the concept of asymptomatic malaria and few were ready to take the drugs in the absence of symptoms. Respondents were likely influenced by the messages from VMWs and other healthcare workers around the judicious use of anti-malarials for symptomatic disease. This indicates potential challenges in achieving uptake of prophylaxis among this group and more targeted research is needed to assess the efficacy, acceptability and feasibility of this approach [3].

Respondents were also familiar with RDTs and placed the confidence in them, with some reportedly bringing RDTs with them to the forest. Respondents also described a readiness to self-treat. Therefore, distributing RDTs for self-testing alongside an ACT for self-treatment after a positive RDT result might appeal to forest workers. This could hasten appropriate treatment for clinical malaria, particularly given the distances that forest workers travel and the extended period they spend away from villages. A similar strategy has been piloted with international travellers who visit malaria endemic areas [32]. Although such a strategy might improve the management of clinical cases, it is unlikely to address malaria transmission maintained by asymptomatic infections and therefore is unlikely to contribute to the elimination of malaria in the GMS [33].

Studying forest work entails questions about and observations of activities that might place respondents in legal jeopardy: many of the forest activities are prohibited, particularly, as in the case in the study area, because they might take place in protected areas, such as national parks. Protecting the personal information of participants in any future research is therefore a priority.

### Strengths and limitations

This is one of relatively few studies that have used qualitative research methods to specifically address malaria-related practices and attitudes of forest workers in the GMS [17]. Using a team of three trained researchers to collect data guarded against the undue influence of a single data collector on the findings. All interviews were conducted in Khmer, including those with respondents with other mother tongues. All respondents spoke Khmer in their daily lives and if unable to adequately express themselves during the interview, assistance was sought from another study team member who spoke the necessary local language and provided translated of specific terms. The data elicited from IDI respondents are limited by the recruitment approach: they all had participated in a clinical trial of malaria treatment in Siem Pang Health Centre. They had, therefore, sought treatment and this approach potentially excluded those unable or unwilling to seek care. Two additional focus groups involved participants recruited from the villages and therefore could have included those unable or unwilling to seek malaria treatment. The similarities between the responses offered by focus group participants from those from interview respondents indicates that this was unlikely to be a source of bias. The findings are drawn from reported data and might be subject to desirability bias, however, for practical reasons, it was not possible to accompany forest workers to undertake direct observations. All respondents were male. This reflects the fact that men are at highest risk for malaria because fewer women engage in forest work. The respondents were ethnically diverse and drawn from a range of villages. Respondents reported multiple bouts of malaria and, even though this group is at high risk, the number of self-reported malaria cases was surprisingly high. Reliable medical records were not available that could be used to link individual participants to proven malaria diagnoses (typically a malaria diagnosis in this local context is an RDT). Although some of these bouts were reportedly confirmed with an RDT, it was not possible to systematically assess whether this was the case for each bout that the respondents described.

## Conclusions

Forest work in Siem Pang District incorporates a diverse set of practices influenced by a range of factors. It is entangled with people’s everyday lives and remains an essential source of income for some. Forest workers are concerned about malaria and try to prevent mosquito bites when in the forest. However, ITNs or hammock nets do not provide protection against outdoor day-time biting vectors. Offering RDT and ACT for self-administration appears acceptable but fails to address the impact of asymptomatic malaria on continued transmission. Respondents were unsure about taking anti-malarials in the absence of symptoms and research to establish the efficacy and feasibility of malaria prophylaxis for forest-goers in the GMS is urgently required.

## Supplementary information

**Additional file 1: Annex S1.** Topic guide for interviews (IDIs and FGDs) with forest workers.

## Data Availability

The data on which this article is based cannot be shared publicly because the informed consent obtained from respondents did not specify that data would be made publicly available and the public availability of data would compromise the privacy of respondents. The data are available upon request to the Mahidol Oxford Tropical Medicine Research Unit Data Access Committee (http://www.tropmedres.ac/data-sharing) complying with the data access policy (http://www.tropmedres.ac/_asset/file/data-sharing-policy-v1-0.pdf) for researchers who meet the criteria for access to confidential data.

## Abbreviations

ACT: artemisinin combination therapy
FGD: focus group discussion
GMS: Greater Mekong Subregion
IDI: individual in-depth interview
IRS: indoor residual spraying
RDT: rapid diagnostic test
VMW: village malaria worker
