# Supplementary material for: Forest work and its implications for malaria elimination: a qualitative study
Source: Malar J. 2019 Nov 27;18:376. doi: 10.1186/s12936-019-3008-3 (PMC6880349; doi:10.1186/s12936-019-3008-3)
Supplement: Supplementary file 1 — Additional file 1: Annex S1. Topic guide for interviews (IDIs and FGDs) with forest workers. [file 12936_2019_3008_MOESM1_ESM.docx]

**Topic guide for interviews (IDIs and FGDs) with forest workers**

Refer to information sheet and training notes and obtain verbal informed consent.

| Topics | Possible probes |
| --- | --- |
| Socio-demographic details | Name  Age  Gender  Village  Employment/livelihood  Languages spoken  Literacy/education (number of school years) |
| Malaria knowledge/exposure | Have you had malaria before?  Where do you think you got malaria from in the past?  Do you know of any malaria hotspots in the area?  Do you think anything you did/didn’t do put you at risk of getting malaria? |
| Forest Behaviour and patterns | How long do people spend at the forest?  Why do people go to the forest?  What do they do there?  How far away is it?  Who do you go to forest with? Do you ever take any family members?  Do you move around in the forest when you visit or do you stay in one place? When do they go there? For how long and how often? (this is critical)  DO you go to different places for different reasons? Please explain…  Do you go to different places at different times of year?  Do you feel at risk of getting malaria when you go to the forest? |
| Sleeping arrangements in forests | Where do you sleep when you are in the forest? Can you describe it (are there walls? overhead coverage? on the floor?)  Do others sleep in the same place?  Do you always sleep in the same place? If not. Where do you move to? Is it the same type of building?  Does everyone use a hammock or sleep under a net?  What about if you go to a new place? |
| Malaria knowledge/exposure | Have you had malaria before?  What were your symptoms?  How did you distinguish malaria from other diseases?  Who diagnosed the disease?  Where do you think you got malaria from in the past?  Do you think anything you did/didn’t do put you at risk of getting malaria?  What did you do to treat the malaria?  Do you know of any malaria hotspots in the area?  Do you think it’s possible to be infected with malaria but not have any symptoms? |
| Health seeking practices | How do you diagnose malaria or high temperature? Do you have a thermometer?  What do you do when you (or a member of your family) have malaria?  Where do they go for assistance when they have malaria?  Do people attend or bypass VMW? Or health centre?  How close is the nearest VMW/health centre (do you have to travel for a long time to get to one)  Do they visit private clinic?  Do people use bednets/hammock nets? Coils? Long sleeves? Or anything else at the forest? If not why? Are any inconvenient or difficult to use?  Do they know where to get nets/hammocks etc.. from?  Where do you get your treatment from? Do you prefer Western or traditional medicine for malaria treatment?  Do you remember reading/seeing any posters or pamphlets explaining how to avoid getting malaria? |
| Experience of antimalarials in the past | What are your experiences of taking antimalarials in the past?  Did you have any side effects?  Would you be willing to take these tablets if you weren’t sick but it could prevent malaria? |
| Experiences of RDTs and other malaria diagnosis | *Do you know what an RDT is?*  *Have you been tested in the past with one? Where did you acquire it or who tested you?*  *Have you ever been tested and get a negative result when you were sick?*  *Do you trust the RDT results to be accurate?* |
| Understanding of vaccination | Have you ever been vaccinated before?  When? What for? Do you think that you will never get that (or other diseases) after being vaccinated? Do you still need to protect yourself from that disease? Has anyone ever explained vaccination to you? If so, who and can you remember what s/he explained? Have you seen posters about vaccination? Do you remember the message of the poster?  Did your children get vaccinated?  Do you prefer injections or medicines to prevent (or cure) diseases? Can you compare these 2 ways? |
